# A molecular 'signature' of primary breast cancer cultures; patterns resembling tumor tissue

**DOI:** 10.1186/1471-2164-5-47

**Published:** 2004-07-19

**Authors:** Shanaz H Dairkee, Youngran Ji, Yong Ben, Dan H Moore, Zhenhang Meng, Stefanie S Jeffrey

**Affiliations:** 1 California Pacific Medical Center, 2330 Clay Street, San Francisco, CA 94115-1932, USA; 2 Department of Surgery, Stanford University School of Medicine, MSLS Building, Room P214, 1201 Welch Road, Stanford, CA 94305-5494, USA

## Abstract

**Background:**

To identify the spectrum of malignant attributes maintained outside the host environment, we have compared global gene expression in primary breast tumors and matched short-term epithelial cultures.

**Results:**

In contrast to immortal cell lines, a characteristic 'limited proliferation' phenotype was observed, which included over expressed genes associated with the *TGFβ *signal transduction pathway, such as *SPARC*, *LOXL1*, *RUNX1*, and *DAPK1*. Underlying this profile was the conspicuous absence of *hTERT *expression and telomerase activity, a significant increase in *TβRII*, its cognate ligand, and the CDK inhibitor, *p21*^*CIP1/WAF1*^. Concurrently, tumor tissue and primary cultures displayed low transcript levels of proliferation-related genes, such as, *TOP2A*, *ANKT*, *RAD51*, *UBE2C*, *CENPA*, *RRM2*, and *PLK*.

**Conclusions:**

Our data demonstrate that commonly used immortal cell lines do not reflect some aspects of tumor biology as closely as primary tumor cell cultures. The gene expression profile of malignant tissue, which is uniquely retained by cells cultured on solid substrates, could facilitate the development and testing of novel molecular targets for breast cancer.

## Background

In breast cancer, cell based experiments are largely conducted with a few spontaneously arising cell lines from late stages of disease that demonstrate unlimited proliferation (immortalization). However, the breadth of tumor heterogeneity and early stages of breast tumorigenesis remain under represented in such assays. We, and others have adapted tissue processing and culture conditions to enable the selective isolation and expansion of tumor cell populations from primary breast cancer [1-9]. Closely reflecting the biological heterogeneity and relatively slower growth rate of malignant cells in primary tumor tissue [10], *in vitro *tumor cell populations are highly variable at the microscopic level and generally require long intervals between passages. The vast majority of such cultures (>90%) display a finite mitotic lifespan. While molecular changes underlying growth cessation in non-malignant breast epithelial cultures are well studied [reviewed in [11]], barriers to the continued proliferation of tumor-derived cultures remain undetermined.

In contradistinction to previous reports on the characteristics of rare immortal cell lines developed from primary tumors [4,5,7,8,12], we have focused on early passage tumor cultures (that may not necessarily culminate as immortal cell lines). This approach encompasses a broader range of disease since <10% of primary breast tumors spontaneously develop into immortal cell lines. Although limited direct comparison of primary breast tumor tissue and corresponding short-term cultures has assisted in authenticating the malignant origin of tumor-derived cultures [1-3,9], relatively little is known regarding the degree of phenotypic and functional concordance between malignant epithelial populations in human breast tissue and their counterparts *in vitro*. In depth comparative analysis of such isogenic malignant cell populations could provide important insights regarding cellular pathways that function independently of environmental constraints, as well as the genome wide consequences of constitutive growth arrest of breast tumor cells widely encountered in laboratory dishes. Here we describe a comprehensive comparison of matched samples of primary breast carcinoma tissue, and early passage tumor cultures. We have used cDNA microarray-based global gene expression profiling to determine common molecular phenotypes of tumor tissue and tumor-derived epithelial cultures, which distinguish them from cancerous and non-cancerous immortal cell lines.

## Results

**Figure 1:**
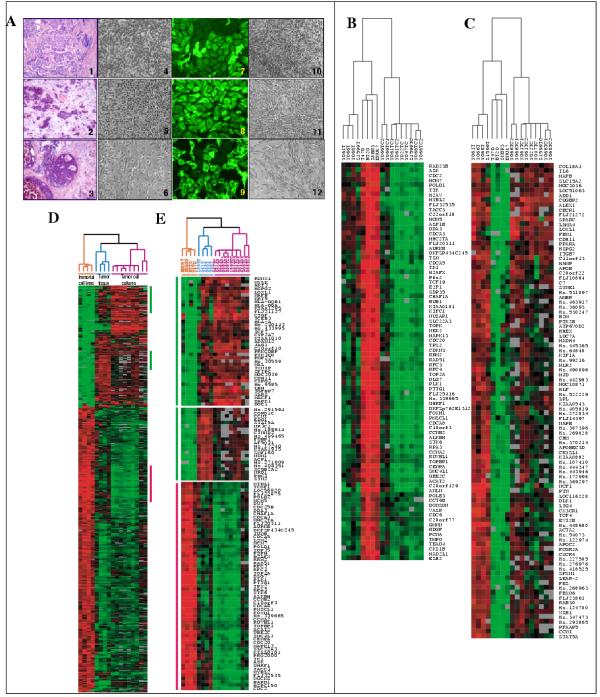
**A. **Microscopic phenotype of primary tumor tissue and corresponding tumor-derived epithelial and stromal cell cultures. (**1–3**) – H & E-stained frozen sections showing histology of representative cases processed for RNA isolation and cell culture. *Case number 061T *– 1; *066T *– 2; *068T *– 3. Note abundant tumor cells in the samples. (**4–6**) – Early passage epithelial cultures; brightfield, 100× magnification. Note morphological variation between cultures in the context of an epithelial phenotype. (**7–9**) – abundant cytokeratin expression in the cytoplasm of cultured primary tumor cells analyzed by indirect immunofluorescence. Magnification – 400×. (**10–12**) – tumor-derived fibroblast cultures; brightfield, 100× magnification. Note morphological distinction between epithelial and stromal cells isolated from the same tissue sample. **B – E. **cDNA-based gene expression profiles of tumor tissue and tumor derived epithelial cultures. Rows represent genes, and columns represent samples. **B, C **– unsupervised two-dimensional hierarchical clustering of 17 samples. **B **– cluster shows genes under expressed in primary tumor tissue and tumor cultures compared to immortal cell lines. **C **– cluster shows genes over expressed in primary tumor cultures and tumor tissue compared to immortal cell lines. **D, E **– Gene expression patterns, which distinguish between group 1, consisting of immortal cell lines, and group 2, consisting of primary tumor tissue and tumor cultures. **D **– Results of SAM analysis showing a thumbnail of 681 differentially expressed genes. **E **– SAM-identified genes which are in common with the cluster in panel B are shown by red vertical bar, and with the cluster in panel C are shown by green vertical bars. Color scale indicates expression level.

In order to ensure the propagation of tumor epithelium without apparent contamination with fibroblasts and other cellular components, previously described methods including the selective release of nests of malignant cells from the connective tissue, were used [2,3]. As illustrated in Figure 1A, the characteristic epithelial morphology, and microscopic heterogeneity between tumor cultures from independent cases was observed. To confirm that these cultures represented pure populations of epithelial cells, indirect cytokeratin immunostaining was performed (Figure 1A). Clinical information and additional cell culture details are listed in Table 1.

**Table 1:** Clinical characteristics of Primary Tumors used for Gene Expression Analysis

**Primary Tumor Cultures**							
**Sample #**	**Sample ID**	**Age**	**TNM Stage**	**Tumor Grade**	**Tumor histology**	**ER status**	**Cell culture passage at RNA isolation**
1	022T	NA	NA	1	IDC	NA	3
2	044T	50	NA	3	IDC	Neg	2
3	047T	79	II	2	IDC	Pos	5
4	054T	59	IV	3	IDC	Pos	8, 12
5	061T*	62	III	2	ILC	Pos	5, 6
6	066T*	47	II	2	IDC	NA	6, 7
7	068T*	52	II	1	IDC	Pos	5, 6
8	071T	NA	II	3	IDC	NA	3
9	076T	51	III	2	IDC	Pos	2
10	257T	41	IIB	3	IDC	Neg	8
11	672T	60	II	3	IDC	Neg	5
12	701T	59	I	1	IDC	Pos	7
13	713T	77	II	3	IDC+ILC	Pos	5, 7
14	1555T	38	NA	2	ILC	NA	3, 4
15	1569T	NA	NA	NA	IDC	NA	3, 4
16	1570T	66	IIA	2	IDC+ILC	Pos	2
17	1599T*	64	I	2	IDC	NA	3
18	1607T	58	I	1	IDC	NA	5
19	1617T	64	III	2	IDC	Pos	2
20	1620T	70	III	3	IDC	Pos	2
21	1625T	80	IIB	3	IDC	Pos	2

* Matched tissue samples analyzed by cDNA microarrays NA – not available; IDC – invasive ductal carcinoma; ILC – invasive lobular carcinoma ER – estrogen receptor; Neg – negative; Pos – positive

### Concordant aspects of global gene expression in breast cancer tissue and tumor-derived cultures

Seventeen RNA samples were analyzed by cDNA microarrays. These were comprised of 4 cases of primary breast tumor tissue and 1–2 matched tumor cultures, 2 additional unmatched tumor cultures, and 4 immortal breast cell lines. Cell lines were selected to represent estrogen receptor (ER) positive (T47D) and ER negative (SKBR3, BT20) tumors, and non-cancerous breast epithelium (ENUt7, ref. [13]).

Unsupervised clustering analysis of 7362 clones (4743 unique genes/ESTs) grouped the samples into three separate clusters representing immortal cell lines, tumor tissue, and tumor cultures. Figure 1B displays a cluster of genes, which were over expressed in immortal cell lines but under expressed in tumor tissue and tumor cultures, while Figure 1C displays a 'mirror image' gene expression pattern (under expressed genes in immortal cell lines, which were over expressed in tumor tissue and tumor cultures). The clusters selected for illustration represent ~90% gene correlation. The entire data set displayed additional clusters [Figure S1 – see  http://genome-www.stanford.edu/breast_cancer/PTCC/].

Comparisons of large data sets as described above frequently result in "significant" patterns of gene expression by chance alone. We employed the Significance Analysis of Microarrays (SAM) for independently verifying genes that are differentially expressed between classes or groups of samples. SAM analysis identified 930 clones, representing 681 unique genes/ESTs, whose expression was significantly (>2-fold) different between group 1 comprised of immortal cell lines, and group 2 comprised of tumor tissue and tumor cultures (0.05% false discovery rate). A full list of the differentially expressed genes is provided in [Table S2 – see http://genome-www.stanford.edu/breast_cancer/PTCC/]. As expected on the basis of the similarity in gene expression observed between tumor tissue and tumor cultures in the unsupervised array data (Figure 1B,1C), these clusters were also present in the SAM profile (Figure 1D). As shown in Figure 1E, genes upregulated in immortal cell lines (indicated by red vertical bar) reflecting significantly shorter doubling times (for example, *RFC4*, *CENPA*, *TOP2A*, *CCNA*, *MCM7*, *PCNA*, *CDC2*) were primarily those associated with the 'proliferation' cluster described by Ross et al [14]. In contrast, upregulated transcripts in tumor tissue and tumor cultures (Figure 1E, indicated by green vertical bar) included genes involved in epithelial differentiation (*MAL, MAFB, RUNX1, KRT5*), in the induction of apoptosis (*DAPK1*), and in tumor angiogenesis, and extravasation (*SPARC*) (Gene Ontology – GO annotations, ref [15]).

Primary epithelial cell cultures, in contrast to fibroblasts and rapidly growing cell lines, undergo rapid growth arrest in 10% fetal calf serum (FCS). This is why we, and others have propagated cultures of primary tumor epithelium in 0–5% FCS (1–9). Immortal cell lines, however, grow optimally in 10% FCS; lower concentrations retard growth. Therefore, to optimize growth conditions for both, 2% FCS was chosen for primary tumor cultures and 10% FCS for cell lines. It is conceivable that an increased concentration of FCS may account for differences in gene expression between primary tumor cultures and immortalized cell lines. In this case, one would expect increased proliferation in the primary tumor cultures at 10% FCS, when in fact, growth is severely inhibited by this approach.

As summarized in Table 2, based on microarray expression data of 38,999 cDNA clones, the average correlation between matched tumor tissue and short-term tumor cultures (7 sets) was 0.41 (sd = 0.03) in contrast to 0.10 (sd = 0.09) between tumor tissue and immortal cell lines (16 pairs). These correlation coefficients differed significantly in a Mann-Whitney rank sum test (p = 0.007) demonstrating that the gene expression profile of tumor tissue was more consistent with that of the corresponding tumor culture than it was with any of the immortal cell lines tested.

**Table 2:** Pair wise correlations between primary tumors, matched epithelial cultures, and immortal breast epithelial cell lines

**Matched Primary Tumor Pair**			**Immortal Breast Epithelial Cell Lines**			
Tissue	Cell Culture		ENUt7	SKBR3	BT20	T47D
***R correlation***						
1599T	1599TC	0.31	0.05	0.07	0.01	0.23
061T	061TC1	0.44	0.10	0.03	0.03	0.24
	061TC2	0.43				
066T	066TC1	0.44	0.16	0.02	0.03	0.20
	066TC2	0.39				
068T	068TC1	0.50	0.12	0.05	0.00	0.27
	068TC2	0.54				

### Determinants of replicative arrest in primary breast tumor-derived cultures

**Figure 2:**
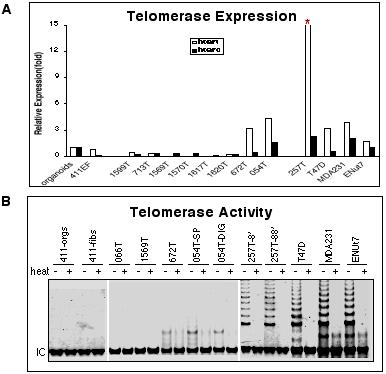
**A. **QRT-PCR analysis of *hTERT *and *hTERC *in primary tumor cultures compared to levels in normal breast organoids, matched fibroblasts, and immortal breast epithelial cell lines (*T47D*, *MDA231*, and *ENUt7*). The Y-axis is minimized to display the range of relative gene expression. The highest *hTERT *expression level, denoted by the asterisk, was 22-fold (sample 257T). **B**. TRAP assay measurement of telomerase activity shown with, and without heat inactivation of cell lysates. Extracts equivalent to 1000 cells are displayed. IC – internal PCR control. For sample 054T, 2 independent fractions of the tumor are shown; SP – mechanically dissociated spillage, DIG – enzymatically digested tissue.

Towards the determination of specific molecular changes underlying the finite proliferative lifespan in tumor cultures, gene expression analysis, by QRT-PCR, was conducted on an expanded set of 39 samples comprised of multiple early passage epithelial cultures obtained from 16 primary breast cancers, 8 cases of normal breast epithelial organoids and, 12 immortal breast epithelial cell lines. As noted above, since genes in the proliferation cluster displayed minimal expression in primary tumor cultures, first we considered the possibility of growth arrest due to a lack of telomerase activity and subsequent telomeric attrition. The relative expression of *hTERC *and *hTERT *subunits of telomerase, encoding the structural RNA component, and the component with reverse transcriptase activity, respectively, was measured. While commonly used immortal cell lines (T47D, MDA231) displayed several-fold higher transcript levels for *hTERT*, undetectable to minimal levels were observed in 6/8 independent primary tumor cultures (1599T, 713T, 1569T, 1570T, 1617T, 1620T). The primary tumor culture with the highest relative expression of *hTERT *(257T) has developed into an immortal cell line (Figure 2A).

To confirm the functional impact of *hTERC *and *hTERT *down regulation, telomerase activity was measured directly by the TRAP assay. As expected, primary cultures with detectable transcript levels, and immortal cell lines of cancerous and non-cancerous origin displayed significant telomerase activity, while those tumor cultures that did not display gene expression showed no activity. In the primary tumor sample, 257T, robust telomerase activity was found as early as passage 8. Similarly, telomerase activity was detectable in early passage epithelial cultures propagated from cells isolated by mechanical dissociation (SP – spillage), or enzymatic digestion (DIG) of the tumor sample 054T (Figure 2B).

**Figure 3:**
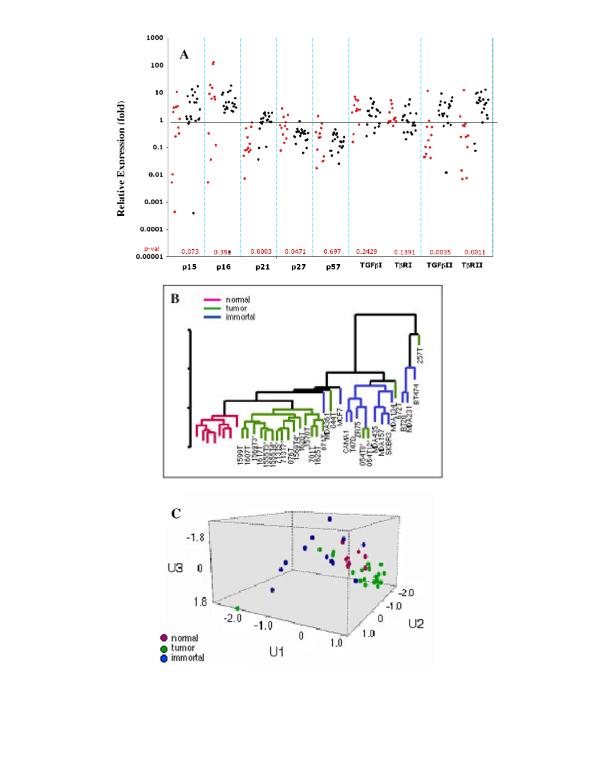
Comparative QRT-PCR analysis of genes encoding negative regulators of cell proliferation in primary tumor cultures and immortal cell lines. **A **– Expression levels of individual genes represented as fold increase or decrease over gene expression in normal breast organoids (average of 8 independent reduction mammoplasty cases). Data shown are averages of triplicate measurements. Dots represent immortal cell lines (red) and primary tumor cultures (black) shown in panels ***B ***and ***C***. **B **– multivariate analysis of data in ***A ***displayed as a hierarchical clustering dendrogram. The scale shows the distance between groups comprised of normal breast, primary tumor cultures, and immortal cell lines. For samples 1569T, 1555T, 713T, and 054T, gene expression was analyzed in epithelial cells at 2 different passages in culture (passage number indicated as 3', 4', 5' etc.) **C **– a plot of 3 principal components (U1, U2, and U3) of 9-dimensional space, representing the 9 genes evaluated in samples in panels ***A ***and ***B***. Based on its level of gene expression, a place is assigned to each cell sample in this space. Data points representing epithelial organoids from the normal breast (pink) appear to be tightly clustered, whereas those representing primary breast tumor cultures (green) and immortal cancer cell lines (blue) show considerable scatter.

In the next step towards identifying the determinants of replicative arrest, primary tumor cultures were compared with immortal cell lines for relative expression of genes associated with the negative regulation of the cell cycle in general, and with epithelial cell proliferation in particular. This analysis included 9 candidate genes in 3 signaling pathways: (1) members of the CIP/KIP family of cyclin-dependent kinase inhibitors (CDKIs), *p21*^*CIP1/WAF1*^, *p27*^*KIP1*^, and *p57*^*KIP2 *^(2) members of the INK family of CDKIs, *p15*^*INK4B*^, and *p16*^*INK4A *^(3) members of the TGF-β family, *TGFβI*, *TGFβII *and the signaling receptors, *TβRI*, and *TβRII*. As illustrated in Figure 3A, we observed that in 10/12 breast cancer cell lines, *p21*^*CIP1/WAF1 *^levels were 2 to138 fold lower than steady state levels in normal breast epithelium. In contrast, 4/20 primary tumor culture samples showed this range of *p21*^*CIP1/WAF1 *^expression (p = 0.0003). For the CDKIs, *p27*^*KIP1*^, and *p57*^*KIP2*^, several fold decrease in gene expression was observed in all primary tumor cultures and most immortal cell lines as well (p = 0.04 and 0.69, respectively). Similarly, no significant differences were apparent for *p15*^*INK4B*^, and *p16*^*INK4A *^gene expression in the two groups (p = 0.07 and 0.39, respectively). For members of the TGF-β family, while significant differences were not observed in the expression of *TGFβI and TβRI*, a median increase of 2 fold and 4 fold were found in the expression of *TGFβII *and *TβRII* respectvely. In contrast, immortal cell lines, showed a median decrease of 7-fold for *TGFβII *and 4-fold for *TβRII *(p = 0.0035 and 0.0011, respectively). All p values were derived by the Mann-Whitney test.

We used MANOVA to compare expression of the 9 above-mentioned genes in immortal cell lines, primary tumor cultures, and normal breast epithelium. It was apparent that the multivariate means for the 9 genes differed for each of the 3 groups above and that both immortal lines and primary tumor cultures differed from normal breast epithelium (p = 0.0001) as also depicted in the hierarchical clustering dendrogram of these samples (Figure 3B). A 3-D plot of the first three principal components, which together account for 83% of the total variation in expression in the 9-dimensional gene space, is shown in Figure 3C. This display format demonstrates that the three types of cell samples cluster in different parts of the three dimensional space and that primary tumor cultures and immortal cell lines are significantly different from normal breast and from each other in the expression of the negative growth regulators evaluated here (p = 0.0002). The relatively large Wilks' lamda (0.271) for immortal cell lines vs. primary tumor cultures is most likely due to the greater spread of these groups in the 9-dimensional gene space, while normal samples are tightly grouped together. This finding may be related to the heterogeneity between individual tumors.

Overall, the genes in the array-based clusters, shown in Figure 1B and 1C, could be categorized as positive or negative regulators of proliferation respectively according to GO annotations. Genes such as, *PCNA*, *CKS1B*, *TPX2*, *UBE2C*, *CDC6*, confirmed by the statistically significant analysis of microarray data, SAM, were among the positive proliferation genes differentially expressed by immortal cell lines, and matched tumor tissue/cell culture samples (Figure 1E, also see Table S2 – http://genome-www.stanford.edu/breast_cancer/PTCC/, for full list). Expression levels of negative proliferation genes identified in the SAM data, such as, *TβRII*, and *CDKN1A *(*p21*^*CIP1/WAF1*^) were confirmed by QRT-PCR (Figure 3A)

## Discussion

The cDNA microarray analysis of primary breast tumors and their epithelial counterparts propagated in cell culture has revealed close similarities in the expression of several hundred genes. Notably, increased expression of genes deemed to be 'growth limiting' by virtue of their consistent down regulation in immortal cell lines, was observed. The patterns of gene up regulation or down regulation were remarkably consistent in independent immortal lines (including those derived from non cancerous breast epithelial cells) as was the contrasting gene 'signature' in cultures derived from independent cases of breast carcinoma. Together, these observations have led us to conclude that the continuous selection of rapidly proliferating cells culminating in the immortalized phenotype favors the loss of several aspects of gene expression retained by early passage primary tumor cultures.

Our data suggests that the limited growth potential of primary tumor cultures results from two major proliferative barriers, (a) telomerase inactivation, potentially leading to telomere attrition, and (b) negative growth signaling by upregulated *TGFβ *resulting in a significant increase in transcription of the CDKI, *p21*^*CIP1/WAF1*^, consistent with the findings of induction or stabilization of this gene in a variety of immortalized cells exposed to exogenous TGFβ [16,17]. Elevated levels of *p21*^*CIP1/WAF1 *^in turn appear to have a direct impact on telomerase regulation [18]. As expected of slow growing primary breast tumors, *in vivo*, p21^CIP1/WAF1 ^positive cells are detected in the majority of cases [19]. Also pertinent in this regard is the fact that substrate induced changes in cell shape upregulate *TGFβ *transcription [20]. Since the promoter region of the *TGFβ *gene contains a shear stress response element [21,22], it seems likely that TGFβ induction occurs in response to unknown stresses in the *in vitro *environment and initiates a cascade of growth inhibitory events, including telomerase inactivation. While epithelial cells derived from most primary breast tumors rapidly revert to regulated growth dictated by environmental signals, immortal breast epithelial cells are insensitive to such cues, which may be the underlying basis for their continued selection *in vitro*. Notably, loss or functional inactivation of the cognate receptor often enables tumor cells *in vivo *to overcome the growth inhibitory effect of TGFβ early in tumorigenesis, however, during metastatic progression, autocrine TGFβ appears to play a tumor-promoting role, possibly enabling tumor cells to survive significant changes in microenvironment [23]. In this light, induction of *TGFβ *in response to the *in vitro *environment in primary breast tumor cultures portrays a critical phase of tumor progression. Additionally, towards the full manifestation of differentiated phenotypes in primary tumor cultures, cell propagation in a three-dimensional growth matrix, pioneered by Bissell and colleagues [24], is imperative. Such studies are currently ongoing in our laboratory.

Tumor-derived immortal cell lines generally display robust proliferation and have thus filled an important need for functional cancer cell model systems. While immortal cell lines continue to provide molecular and biological insights regarding proliferation-related hallmarks of malignancy, their functional application as indicators of efficacy in cancer drug development is relevant mostly to rapidly proliferating high-grade tumors. Many breast tumors do not fall into this category, and are not necessarily indolent. Thus, additional targets in diverse gene clusters must be identified for novel drug designing. In fact, such targets could be applicable to tumors at early or late stages as recent studies suggest that genes conferring invasive and/or metastatic characteristics late in progression often become dysfunctional at earlier stages of tumorigenesis [25]. Moreover, since our data demonstrate that primary tumor cultures routinely derived from surgical discard tissue display phenotypic, and most likely functional aspects of breast cancer, they provide a strong rationale for the experimental manipulation of such cells towards revealing the causative role of genetic polymorphisms in cancer susceptibility, an important goal which is unlikely to be fulfilled with currently used model systems.

## Conclusions

This microarray-based analysis demonstrates that epithelial cultures isolated from primary breast tumors retain phenotypes of the malignant tissue, which are eliminated during the selection of rapidly proliferating cell populations that comprise commonly used *in vitro *model systems. Thus the opportunity for basic and clinical application of functional cells derived from the full range of pathological breast tissue, instead of a few immortal cell lines should not be missed.

## Methods

### Clinical specimens and cell culture

Pathologically confirmed tumor tissue and non malignant reduction mammoplasty samples were collected as fresh specimens under IRB approved guidelines at the California Pacific Medical Center, San Francisco, Stanford University, and the University of California, San Francisco between 1997 – 2000. Additional tumor samples were obtained from the NCI Cooperative Human Tissue Network. A portion of the tissue was snap frozen and cryopreserved for histological confirmation and nucleic acid isolation prior to cell culture. Tumor samples were processed as previously described [2,3]. A total of 21 independent cultured specimens were used for gene expression analysis. Primary tumor cultures and the non-tumorigenic, *ENUt7 *cell line were propagated in low calcium MCDB170 medium supplemented with 2% FCS. Breast cancer cell lines were cultured in the recommended growth media supplemented with 10%FCS; *MCF7*, *BT20*, *MDA231*, *MDA157 *in DME-H21; *BT474, CAMA1*, *T47D*, *ZR75 *in RPMI 1740; *MDA435*, *MDA134*, *MDA361 *in L15.

To confirm the epithelial phenotype of primary tumor derived cells, cultures fixed with 50% ethanol: acetone were immunostained with anti pan-cytokeratin, as previously described [1].

### RNA isolation, microarray methods and data analysis

Frozen tissue was trimmed to yield >90% tumor cell enrichment. Total RNA was extracted with the RNAeasy Mini kit (Qiagen), amplified using an optimized T7 based protocol [26], labeled with Cy5, and hybridized to ~47,000 feature cDNA microarrays as previously described [26,27]. Hybridized arrays were scanned (Axon) and images were analyzed using GenePix^® ^Pro 4.0 software (Axon Instruments). Spots were selected for analysis if signal intensities in both Cy3 and Cy5 channels were 2.5 times higher than background. The arrays were clustered using a hierarchical clustering algorithm [28], which groups genes and samples on the basis of their expression similarities. The results were visualized using TreeView software [29]. The clustering was performed on clones whose expression varied at least 3-fold from the mean in one or more samples and was measurable in over 80% of the samples.

Significance Analysis of Microarrays [30,31], used for identifying genes whose expression differed significantly between groups, was performed on data from 38,999 cDNA clones. After the selection of spots 2.5 times over background, and the elimination of clone redundancy, data was retrieved on 930 clones, representing 681 unique genes.

### Quantitative Real Time PCR (QRT-PCR)

RNA samples were treated with RNAse-free DNAse (Roche) to remove genomic DNA, and reverse transcribed. Fifty ng of cDNA was used as template for PCR amplification with specific oligonucleotide primers, (designed using Primer Express 1.5 software). Following denaturation and cycling reactions (40 cycles) the products were analyzed (Applied Biosystems 5700 Sequence Detection System). The cycle number at which the amount of amplified target reached a fixed threshold was designated as the threshold cycle (C_T_). The higher the initial amount of transcript template, the lower the C_T _value. For quantitation of gene expression, the target gene value normalized to the expression of an endogenous reference (*β actin*) was designated as ΔC_T_. Relative gene expression was calculated by the formula 2^-ΔΔCT^. ΔΔC_T _was obtained by subtracting the ΔC_T _of test sample from the ΔC_T _of normal breast epithelial fractions, called 'organoids', isolated from mechanically and enzymatically dissociated reduction mammoplasty tissue (average of 8 independent specimens).

### Telomerase activity

Cell lysates prepared from primary tumor cultures, immortal cell lines, and normal breast organoids were analyzed by the telomerase repeat amplification protocol (TRAP) as per manufacturer recommendation (Intergen). Briefly, trypsinized cell pellets were resuspended in lysis buffer at 500 cells/microliter, incubated on ice for 30 minutes, centrifuged at 12,000 × g for 20 mins, and the telomerase containing supernatant used for PCR amplification. Reaction products were resolved in 10% PAGE gels and visualized with SYBR Green-1 (Molecular Probes). Heat inactivated controls were included for each sample. Linearity of the assay was determined with cell dilutions ranging from 100 to 10, 000 cells.

### Statistical methods

We calculated Pearson correlations from the microarray data generated from 38,999 clones to measure similarities among 7 matched tumor tissue: cell culture sample pairs and among 16 tumor tissue: immortal cell line pairs. For QRT-PCR data, multivariate analysis of variance (MANOVA) was used to compare the expression levels of genes involved in negative growth regulation between immortal cell lines, primary tumor cultures, and non malignant breast epithelial cells. Tests for statistical significance were based on Wilks lamda criterion, a multivariate analog of the F-test for univariate analysis of variance (ANOVA), which tests the equality of means. Calculations were made in Data Desk, version 6.2. Principal components for all genes over all the data were computed and S-Plus was used to plot the data for the first 3 principal components.

## Authors' contributions

SHD conceived the study, provided overall direction and coordination to the research, and drafted the manuscript. YJ carried out the microarray analysis. YB carried out QRT-PCR, and other molecular analyses. DHM performed the statistical analysis of data. ZM performed the pathology review and tissue dissection. SSJ participated in the design and analysis of microarray-based experiments and in editing portions of the manuscript. All authors read and approved the final manuscript.
